# HIV-1 Gag-protease-driven replicative capacity influences T-cell metabolism, cytokine induction, and viral cell-to-cell spread

**DOI:** 10.1128/mbio.03565-24

**Published:** 2025-02-25

**Authors:** Omolara O. Baiyegunhi, Kensane Mthembu, Ann-Kathrin Reuschl, Doty Ojwach, Omotayo Farinre, Murunwa Maimela, Sheila Balinda, Matt Price, Madeleine J. Bunders, Marcus Altfeld, Clare Jolly, Jaclyn Mann, Thumbi Ndung’u

**Affiliations:** 1 Africa Health Research Institute 145749 , Durban, South Africa; 2 HIV Pathogenesis Programme, The Doris Duke Medical Research Institute, University of KwaZulu-Natal, Durban, South Africa; 3 Division of Infection and Immunity, University College London, London, United Kingdom; 4 Medical Research Council, UVRI & LSTHM Uganda Research Unit, Entebbe, Uganda; 5 Department of Epidemiology and Biostatistics, University of California San Francisco, San Francisco, California, USA; 6 IAVI, New York, New York, USA; 7 Division of Regenerative Medicine and Immunology, III. Department of Medicine, University Medical Center Hamburg-Eppendorf, Hamburg, Germany; 8 Ragon Institute of MGH, MIT, and Harvard University, Boston, USA; 9 Research Department Virus Immunology, Leibniz Institute of Virology 28367 , Hamburg, Germany; The University of North Carolina at Chapel Hill School of Medicine, Chapel Hill, North Carolina, USA

**Keywords:** HIV, Gag, replicative capacity, T-cell, metabolism, subtypes, inflammation, cell-to-cell spread

## Abstract

High replicative capacity (RC) HIV-1 strains are associated with elevated viral loads and faster disease progression in the absence of antiretroviral therapy. Understanding the mechanisms by which high RC strains adversely affect the host is essential for developing novel anti-HIV interventions. This study investigates cellular metabolism, cytokine induction, and cell-to-cell spread as potential mechanisms differentiating clinical outcomes between low and high RC strains of HIV-1. We constructed chimeric viruses containing patient-derived *gag-proteases* from HIV-1 subtypes B and C in the NL4-3 backbone. Viral RC was determined using a green fluorescent protein (GFP)-reporter T-cell line assay and cytokine production in T-cells was assessed using Luminex. Virus cell-to-cell spread efficiency was measured through flow cytometry-based detection of p24, while nutrient uptake assays and mitotracker dye detection served as surrogate markers for T-cell metabolism and mitochondrial function. Chimeric subtype C viruses exhibited significantly lower RC compared to subtype B viruses (*P* = 0.0008). Cytokine profiling revealed distinct cytokine signatures associated with low RC subtype C viruses. Viral RC negatively correlated with tumor necrosis factor alpha (TNF-α), IL-8, and IL-13 induction, while it positively correlated with platelet-derived growth factor (PDGF-bb), IL-7, monocyte chemoattractant protein-1 (MCP-1), fibroblast growth factor (FGF)-basic levels, viral spread efficiency (*P* = 0.008, *r* = 0.5), and cellular glucose uptake (*P* = 0.02, *r* = 0.5). Conversely, RC was negatively correlated with glutamine levels (*P* = 0.001, *r* = −0.7), indicating a link between RC and nutrient utilization. Furthermore, mitochondrial depolarization was elevated in subtype B infections when compared to subtype C infections (*P* = 0.0008). These findings indicate that high RC strains exert distinct cellular effects that may influence HIV-1 pathogenesis, highlighting the need to develop novel therapeutic strategies.

**IMPORTANCE:**

Virus replicative capacity (RC) influences disease progression following HIV-1 transmission; however, the mechanisms underlying the differential clinical outcomes remain poorly understood. Our study reveals variations in cytokine induction and cellular metabolism in T-cells infected with HIV-1 subtype B and C viruses exhibiting high or low RC. T-cells infected with high RC strains showed increased induction of IL-7 and platelet-derived growth factor (PDGF-bb), along with heightened glucose uptake and elevated glutamine consumption compared to those infected with low RC strains. By contrast, low RC strains induced higher levels of IL-8, IL-13, and tumor necrosis factor alpha (TNF-α) and demonstrated reduced efficiency in modulating cellular metabolism and virus cell-to-cell spreadability. These findings highlight distinct biological differences between high and low RC viruses, providing valuable insights into the mechanisms that may underpin varying clinical outcomes. This knowledge may inform the development of novel interventions aimed at limiting viral virulence or transmission.

## INTRODUCTION

The extreme viral genetic diversity of HIV-1 poses a challenge for designing immune-based prophylactic and treatment strategies. HIV-1 group M, which is responsible for most global HIV-1 infections, comprises several phylogenetically distinct subtypes with unique geographic distributions (1). Subtype B is prevalent in Europe and North America, while subtype C infections predominates in southern Africa, Ethiopia, Brazil, and India; subtypes A, D, and their recombinants are found in East Africa, while West Africa and the Congo Basin exhibit diverse and phylogenetically complex epidemics (2, 3). Understanding the biological characteristics of different HIV-1 subtypes is crucial for advancing HIV-1 research and developing novel biomedical interventions.

HIV-1 replicative capacity (RC) is an *in vitro* measure of virus fitness within a host and generally correlates with viral loads and disease progression (4–6). Moreover, *in vivo* viral fitness is influenced by immune or drug pressure, meaning lower RC may confer an advantage in certain contexts (7). However, the mechanisms by which RC or subtype-specific differences influence pathogenesis remain poorly understood. HIV-1 infection disrupts immune signaling pathways, leading to a cytokine storm during acute infection that drives immune dysregulation and viral replication (8–10). Studies indicate that high RC viruses induce elevated cytokine levels (11), but the impact of RC on cytokine production in specific immune cells like CD4^+^ T-cells, remains underexplored. Cellular metabolism influences cytokine signaling pathways, impacting immune cell function (12). HIV-1 infection induces metabolic reprogramming in CD4^+^ T-cells, which supports viral replication and immune cell functions such as activation and differentiation (13, 14).

In this study, we investigated the mechanisms influencing the spread of viruses with varying replicative capacities or viral subtypes in CD4^+^ T-cells. We examined how RC and viral subtypes affect cytokine production, cellular metabolism, and virus cell-to-cell spread in CD4^+^ T-cells, hypothesizing that high RC viruses would drive greater inflammatory responses, metabolic changes, and increased cell-to-cell spread efficiency. Using *in vitro* T-cell models we assessed 27 chimeric NL4-3 viruses containing patient-derived HIV-1 *gag-proteases* from subtypes B and C.

## RESULTS

### Chimeric HIV-1 viruses of subtype C have lower replicative capacities than subtype B viruses

**Fig 1:**
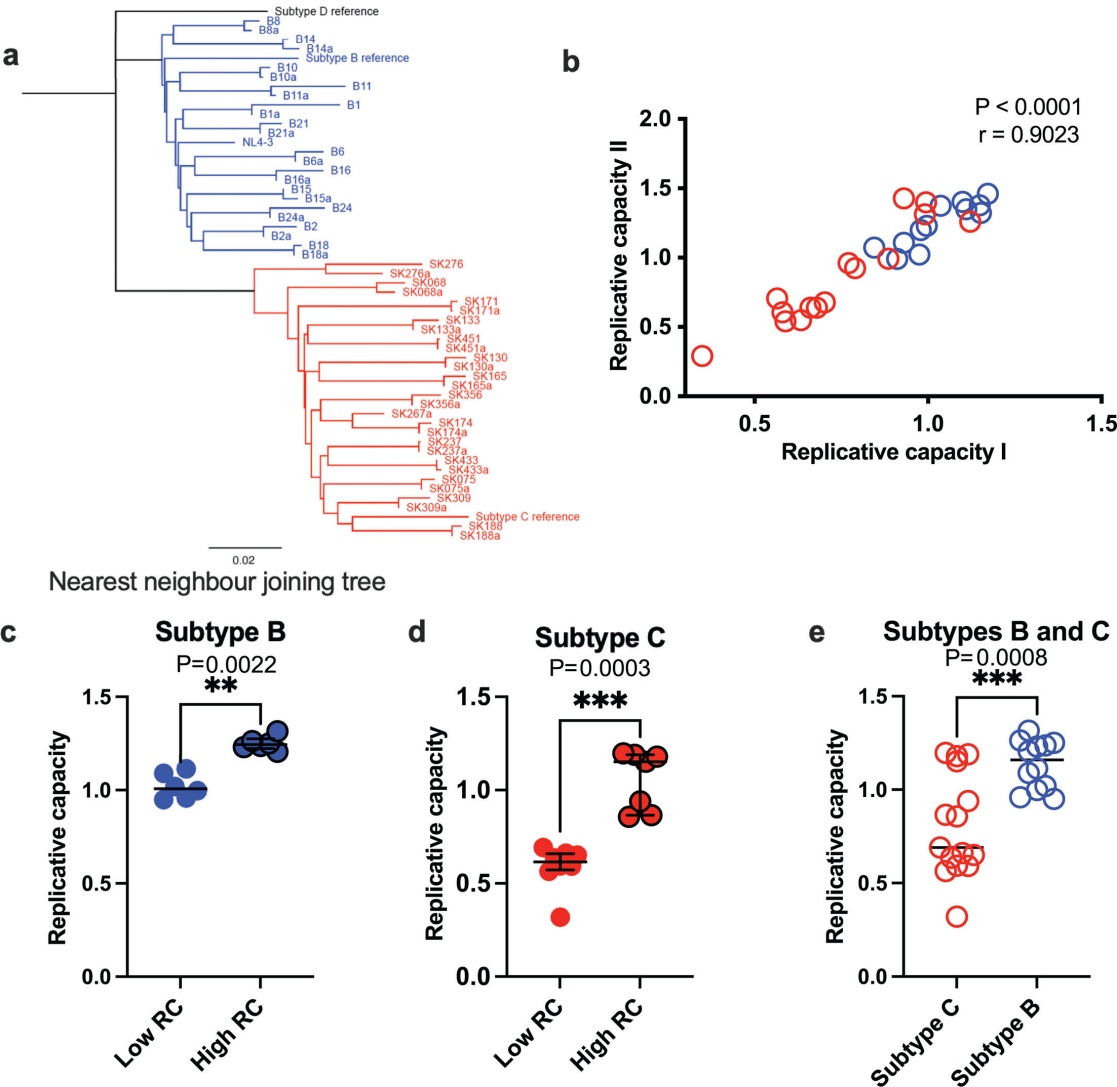
Phylogeny and RCs of HIV-1 subtypes B and C. (**a**) Maximum-likelihood phylogenetic tree of *gag-protease* sequences from cohorts in South Africa (subtype C) and Canada (subtype B). Sequences generated for this study are paired with sequences from GenBank, denoted with the suffix ”a,” except for one participant whose sequence was not available. The reference sequences for different HIV-1 subtypes were obtained from the HIV sequence database (https://www.hiv.lanl.gov/content/index). (**b**) Correlation between two independent RC experiments. (**c and d**) Comparison of RCs for HIV-1 subtype B (**c**), subtype C (**d**), and a combined analysis of low- and high-RC viruses within subtypes B and C chimeric viruses (**e**). (**b–e**) Each dot represents one virus, with the median line shown for each data set. All statistical tests are two-sided. Two-tailed *P* values from the Mann-Whitney U test (**c and d**) are displayed. Spearman’s rho (*r*) values and *P* values are reported for the correlation analyses (**b**).

Our study was designed to investigate cellular metabolic differences, cytokine induction, and cell-to-cell spread between low RC and high RC viruses from HIV-1 group M subtypes B and C. We analyzed 27 chimeric viruses derived from participants previously characterized within chronic HIV-1 infection cohorts in Canada and South Africa (5, 15). Virus stocks were generated for each sample, and sequencing confirmed that the *gag-protease* regions and viral subtypes aligned with prior classifications (Fig. 1a). Replicative capacity assays yielded highly concordant results across two independent experiments (Fig. 1b). As expected, the RC values reflected both low RC and high RC viruses in subtypes B and C (Fig. 1c and d), with subtype B viruses exhibiting significantly higher RCs than subtype C (Fig. 1e). These results are consistent with the previously reported differences in RC between subtypes B and C viruses (15).

### Influence of virus RC and subtype on cytokine expression

**Fig 2:**
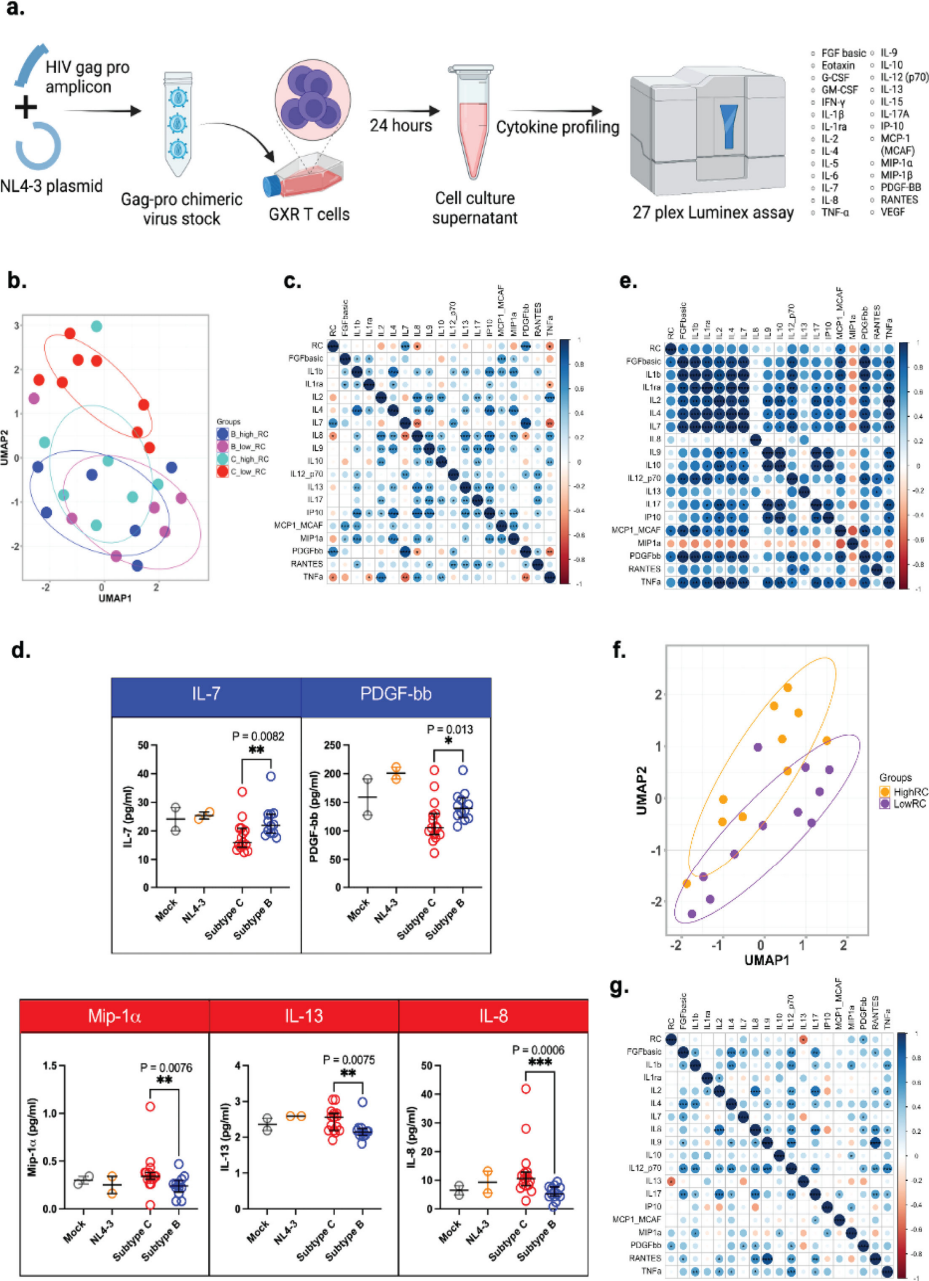
Impact of HIV-1 Gag-protease mediated RCs on cytokine expression *in vitr*o and in plasma from acute HIV-1 infection. (**a**) Schematic of *in vitro* infectivity experiment for cytokine profiling in GXR-T cells. (**b**) Uniform manifold approximation and projection (UMAP) analysis of cytokine expression in GXR-T cell lines across virus RC groups. (**c**) Spearman correlation matrix showing the relationship between virus RCs and cytokines expressed by GXR-T cell lines across all viruses. (**d**) Comparison of cytokines induced by infections with HIV-1 subtypes B and C viruses in GXR-T cells. (**e**) Spearman correlation matrix illustrating the relationship between viral RCs and cytokine expression in primary CD4^+^ T cells, comparing eight selected subtypes B and C viruses. (**f**) UMAP analysis of plasma cytokine expression in low and high RC groups from an HIV-1 subtype C infection cohort. (**g**) Spearman correlation matrix showing the correlation between plasma cytokine levels and virus RC values. All statistical tests are two-sided. Mann-Whitney U test *P* values are shown in panel d and Spearman coefficients are depicted by the color key in the correlation matrices (**c, e, g**), with corresponding *P* values. *P < 0.05, **P < 0.01, ***P < 0.001, ****P < 0.0001.

To determine whether virus RC or subtype influences the induction of specific immune-modulatory cytokines, we measured a panel of 27 cytokines in the supernatants of infected CEM-GXR T-cells (Fig. 2a). Cytokine profiling revealed that low RC subtype C viruses formed a distinct cluster (Fig. 2b). Notably, IL-7 and platelet-derived growth factor (PDGF-bb) levels showed a significant positive correlation with virus RC, while IL-8 and tumor necrosis factor alpha (TNF-α) correlated negatively with RC (Fig. 2c). Further analysis by viral subtype indicated that IL-7 and PDGF-bb were significantly elevated in subtype B infections compared to subtype C (Fig. 2d). By contrast, subtype C induced significantly more macrophage inflammatory protein alpha (MIP-1α), IL-13, and IL-8 than subtype B (Fig. 2d). In addition, in subtype C infections, IL-7, PDGF-bb, fibroblast growth factor (FGF)-basic, and monocyte chemoattractant protein-1 (MCP-1) were significantly induced by viruses with high RC compared to those with low RC (Fig. S1a). However, cytokine expression for some viral infections overlapped with the controls (Fig. 2d; Fig. S1a).

Cytokine induction was also assessed in primary CD4^+^ T cells using a subset of three subtype B viruses and five subtype C viruses, selected based on sample availability. In these primary cells, levels of PDGF-bb, FGF-basic, and MCP-1 positively correlated with viral RC (Fig. 2e). Moreover, we observed a trend toward higher levels of IL-7, PDGF-bb, FGF-basic, and MCP-1 in subtype B compared to subtype C infections, consistent with the findings from GXR-T cells (Fig. S1b). These results highlight RC- and subtype-specific differences in the induction of immune-modulatory cytokines.

### Validation of cytokine signatures in high and low RC virus infections using primary plasma samples from acute HIV-1 infection

To further understand the role of cytokines produced during primary infection, we analyzed archived plasma samples from an available subtype C HIV-1 infection cohort. These samples had previously defined virus RC data (Table 2; Fig. S1c). Cytokine expression partially clustered the high and low RC groups (Fig. 2f). Specifically, PDGF-bb levels correlated positively with RC while IL-13 showed a negative correlation (Fig. 2g). Moreover, PDGF-bb and IL-7 levels were higher in the high RC group (Fig. S1d).

### Chimeric viruses with high replicative capacity exhibit greater efficiency in cell-to-cell spread

**Fig 3:**
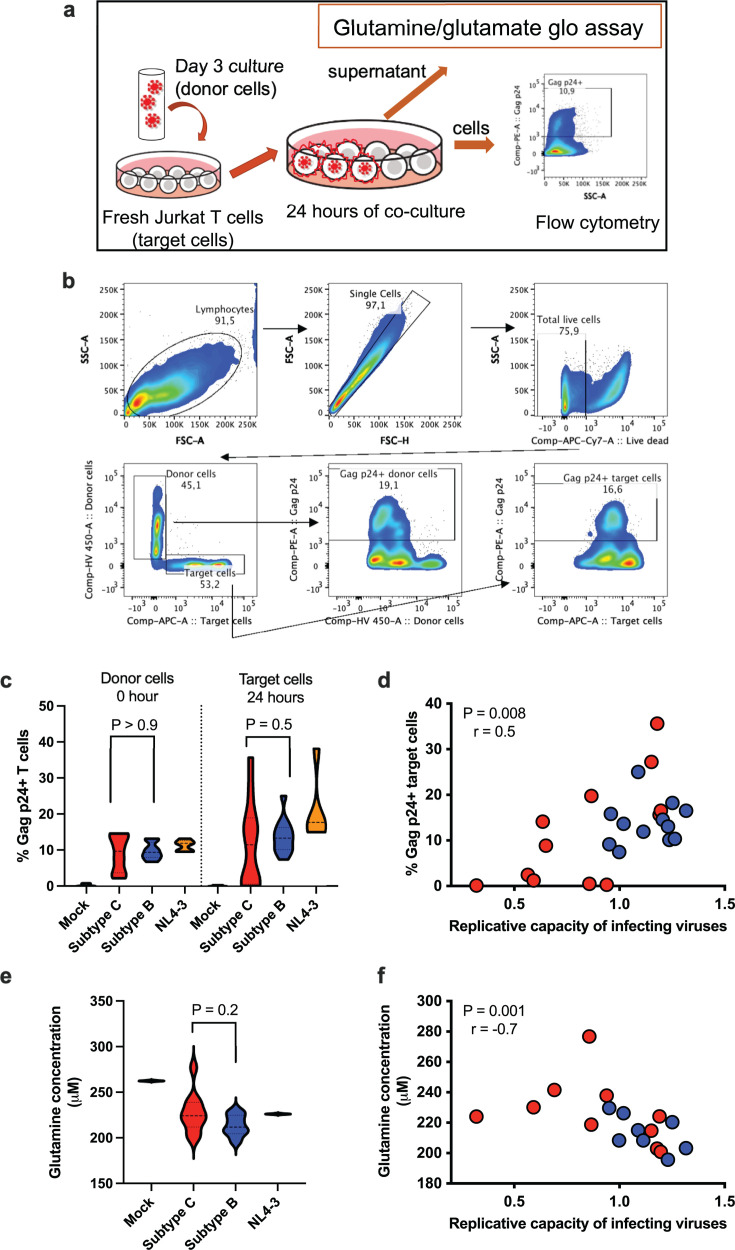
HIV-1 Gag-protease mediated RCs correlate with the virus’s ability for cell-to-cell spread and amino acid consumption in Jurkat cell lines. (**a**) Schematic of cell-to-cell spread assay. (**b**) Flow cytometry gating strategy for Gag p24-positive target cells. (**c**) Violin plots showing the frequency of Gag p24-positive donor cells at the start (0 hours) and of target cells after 24 hours of co-culture with donor cells. (**d**) Correlation analysis of virus RCs and cell-to-cell spread capability. (**e**) Glutamine levels in virus-infected cell culture supernatants after 24 hours. (**f**) Correlation analysis of virus RCs and glutamine measurements (*n* = 18 viruses). All statistical tests are two-sided. Spearman rho (*r*) values and *P* values are reported for the correlation analyses (**d and f**).

Cell-to-cell spread is a predominant mode of HIV-1 transmission (16–19), but the viral determinants regulating the efficiency of cell-to-cell spread remain incompletely characterized. Given the important influence of Gag in RC (11, 15) and its role in co-operating with Env during cell-to-cell spread (20), we investigated the relationship between Gag RC and cell-to-cell spread efficiency. To do this, we compared the capacity of high and low RC viruses from HIV-1 subtypes B and C to disseminate between T cells via cell-to-cell spread (experimental design depicted in Fig. 3a). Flow cytometry analysis of Gag p24-positive target cells after 24 hours of co-culture with infected donor T cells revealed differences in the efficiency of cell-to-cell spread for all viruses tested (Fig. 3b and c; Fig. S2a). Low RC subtype C viruses in particular exhibited lower infection rates (Fig. S2a). In addition, we observed a positive correlation between viral RC and the efficiency of cell-to-cell spread (Fig. 3d; Fig. S2b).

Given glutamine’s role in supporting HIV-1 replication (21), we investigated whether cell-to-cell viral spread is associated with glutamine utilization across 18 samples. Glutamine levels showed a hierarchical distribution according to RC groups and subtypes (Fig. S2c; Fig. 3e). Notably, a significant negative correlation was observed between glutamine levels and virus RC (Fig. 3f), suggesting increased glutamine consumption in high RC cultures, which resulted in lower glutamine levels. These findings suggest that virus RC may influence nutrient utilization within infection cultures.

### Cellular glucose uptake, but not fatty acid uptake, correlates with the RC of infecting viruses

**Fig 4:**
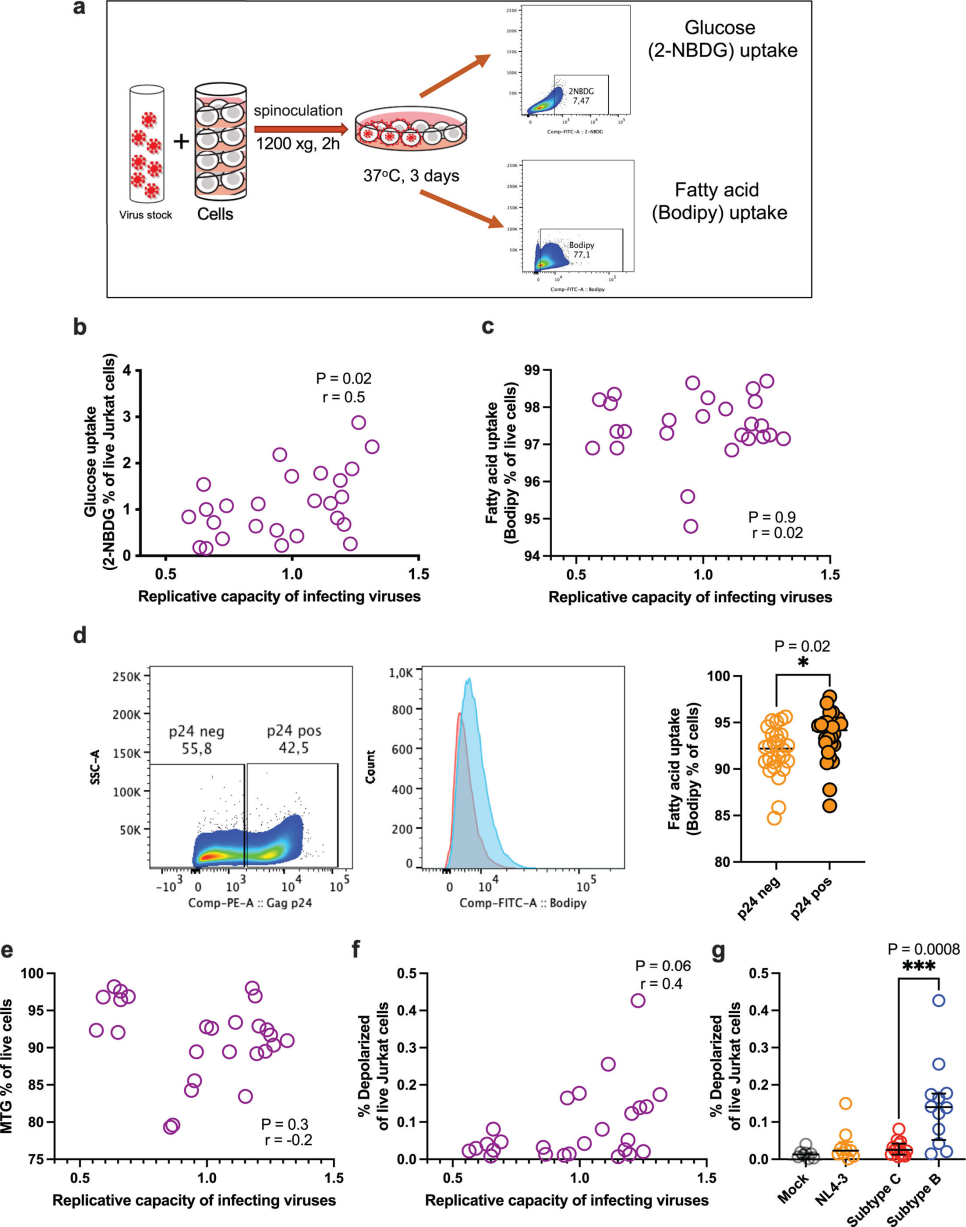
Correlation of HIV-1 Gag-protease mediated RCs with nutrient uptake and mitochondrial function in *in vitro* infected Jurkat cells. (**a**) Schematic of the metabolic uptake assay. (b and c) Aggregate data showing the correlation between virus RCs and glucose uptake (**b**) or fatty acid uptake (**c**). (**d**) Representative flow cytometry gating and summary data of fatty acid uptake in infected (p24 positive; pos) and bystander uninfected (p24 negative; neg) cells. (**e**) Correlation analysis between virus RCs and mitochondrial mass, measured as the percentage of Mitotracker Green (MTG)-positive cells. (**f**) Correlation analysis between virus RCs and mitochondrial membrane depolarization (MMD). (**g**) Summary data comparing MMD across subtype groups. All statistical tests are two-sided. Two-tailed *P* values from the Mann-Whitney U test (**d and g**) are displayed. Spearman’s rho (*r*) values and *P* values are reported for correlation analyses (**b, c, e, f**).

We further investigated whether the uptake of other nutrients, specifically glucose and fatty acids, was modulated in infections with low RC or high RC viruses. The uptake of 2-[N-(7-nitrobenz-2-oxa-1,3-diazol-4-yl)amino]-2-deoxy-D-glucose (2-NBDG), a fluorescent marker for glucose uptake, is commonly used as a proxy for cellular glucose consumption (22, 23). We measured glucose uptake in Jurkat cells spinoculated with the virus after 72 hours post-infection (experimental schematic shown in Fig. 4a). Our results demonstrated that glucose uptake was up to twofold lower in cultures infected with low RC subtype C viruses compared to those infected with other viruses (Fig. S3a). There was a modest positive correlation between glucose uptake and virus RC (Fig. 4b), indicating that high RC viruses induce greater glucose uptake.

In contrast, flow cytometric detection of Bodipy, a dye used to measure fatty acid uptake, showed high uptake across all cultures but no correlation with either virus RC or subtype (Fig. 4c; Fig. S3b). When we distinguished productively infected (Gag p24-positive) cells from bystander uninfected cells, we found that infected cells exhibited elevated fatty acid uptake compared to uninfected cells within the same culture (Fig. 4d; Fig. S3c and d).

Given that mitochondria function as energy production hubs upon nutrient uptake, we next assessed mitochondria mass and depolarization to evaluate the impact of virus RC on mitochondrial function. No significant correlation was observed between mitochondria mass and overall RC or subtype (Fig. 4e; Fig. S3e and f). However, the frequency of depolarized mitochondria moderately correlated with virus RC (Fig. 4f) and was highest in subtype B infections compared to subtype C infections (Fig. 4g; Fig. S3g), suggesting that high RC infections may lead to impaired mitochondrial function.

## DISCUSSION

This study offers novel insights into how HIV-1 Gag-protease-driven replicative capacity (RC) influences cellular cytokine production, T-cell metabolism, and virus cell-to-cell spread. We previously demonstrated the compatibility of the subtype B NL4-3 backbone with subtype C Gag-proteases (5, 15), allowing us to generate chimeric viruses with patient-derived Gag-proteases inserted into the same viral backbone. Importantly when Gag-proteases from diverse subtypes are cloned into a subtype C backbone (15), a similar subtype hierarchy of RCs is observed as in the NL4-3 subtype B backbone indicating that the backbone may not substantially impact RC data. The findings for HIV-1 Gag-protease chimera may, however, not fully represent the behavior of the full virus or its other proteins, because although the correlation between Gag-protease viral RC and the RC of whole virus isolates was statistically significant, it was a weak correlation (15).

Our findings reveal that high RC viruses exhibit higher cell-to-cell spread, increased glucose uptake, and glutamine consumption, alongside associated mitochondrial dysfunction and elevated induction of IL-7, PDGF-bb, MCP-1, and FGF-basic. We also noted subtype-specific differences in how chimeric viruses modulate cellular responses, which may impact antiviral immunity and inflammation. Specifically, subtype C infections are characterized by lower virus RC, but higher levels of MIP-1α, IL-13, and IL-8, along with lower frequencies of depolarized mitochondria, compared to subtype B infections.

Virus-host interactions during early HIV-1 infection elicit diverse immune responses, including the expression of restriction factors and cytokine signaling pathways. Focusing on CD4^+^ T-cells—primary HIV-1 targets and key drivers of persistent immune activation and inflammation (24–26)—we observed that low RC infections produced elevated levels of pro-inflammatory cytokines, such as TNF-α, and IL-8 which negatively correlated with virus RC. TNF-α induces IL-8 expression in response to certain bacterial infections (27), correlating with the activation of NF-kB and P13-Akt pathways, crucial for HIV-1 transcription and replication (28). The induction of these key intermediates by low RC viruses may be consequential to the survival of infected cells to allow for full virus replication cycles and drive viral propagation in the long term. Notably, MCP-1, which signals downstream to PDGF-bb (29–31), was higher in high RC infections. HIV-1-mediated induction of PDGF-bb occurs through the c-Jun N-terminal kinase (JNK) pathways (31). Elevated levels of PDGF-bb, and MCP-1 may promote apoptosis via the JNK pathways, although further studies are needed to confirm this.

Our findings contrast with previous research on acute HIV-1 infection, particularly regarding the correlation between plasma cytokines and viral RC. Previous studies, including our own, reported high induction of IL-1β, IL-6, IL-10, and IP-10, by high RC viruses (8, 11). However, this study directly measures the impact of HIV-1 infection on T-cell lines or primary CD4^+^ T cells *in vitro,* contrasting with plasma cytokine measures in prior studies that encompass contributions from diverse immune cell types. Validation from acute HIV-1 plasma samples revealed elevated levels of IL-7 and PDGF-bb in the high RC group, consistent with some earlier findings (11). The differences between our study and previous work may stem from the timing of infection. Longitudinal analysis of acute infections indicates variable timing of peak cytokine expression (8). For instance, MCP-1, IL-8, and interferon-γ (IFN-γ), peak by the second week and resolve by the fourth week, while others peak later with partial resolution (8). Here we analyzed plasma cytokines later in acute infection, possibly after the resolution of the previously identified cytokine storms.

Previous research has shown that IL-7 stimulation of T-cells prior to *in vitro* HIV-1 infection, enhances glucose uptake and T-cell permissivity to HIV-1, accompanied by increased expression of the GLUT-1 receptor responsible for glucose transport (32). Although we did not measure the GLUT-1 receptor in our study, we observed elevated glucose uptake in high RC virus infections. One hypothesis is that higher glucose uptake in high RC infections drives the elevated cytokine levels observed. High cellular glucose concentrations have been linked to increased expression levels and enhanced binding affinity of the PDGF-bb receptor (33). Further research is needed to explore the effect of these cytokines on HIV-1 pathogenesis and the high RC HIV-1-induced reprogramming of glucose uptake, as this could inform new therapeutic interventions.

Viruses with higher RC demonstrated greater efficiency in cell-to-cell spread, aligning with previous findings on the significance of this mode of HIV-1 replication (16–19, 34). This enhanced spread may influence host responses, particularly cytokine production and nutrient metabolism, including the well-documented dysregulation of glutamine metabolism during HIV-1 infection to meet the increased demand for essential components required for virion assembly (35, 36). Given the central role of mitochondria in cellular metabolism (37), impaired mitochondrial function could adversely affect cellular operations. Further experiments such as the Seahorse assay, which directly measures mitochondrial function, could provide deeper insights into potential impairments associated with high RC infections. Future studies should integrate the various virus RC-associated pathogenesis pathways identified in this study to develop a more comprehensive understanding of their impact.

Our study has some limitations that warrant acknowledgment. Firstly, we utilized Jurkat T-cell lines, which, while advantageous for reproducibility, may not fully recapitulate the function of primary T-cells. Secondly, our focus on Gag-protease function, while correlated with the RC of the whole virus (11, 15), excludes the contribution of other essential HIV-1 proteins. Therefore, our findings regarding the Gag-proteases of subtype B and C viruses may not fully represent the behavior of the complete virus. Additionally, examining other viral genes/proteins or full-length subtype B or C viruses could yield different results. While introducing subtype C Gag-protease into the subtype B NL4-3 viral backbone might disrupt the natural context, our previous findings suggest otherwise (15). Moreover, the cellular processes studied may vary over time, and our snapshot observations at specific time points post-infection in an *in vitro* system may not fully reflect the dynamic cellular immune interactions *in vivo*.

In conclusion, our study elucidates that HIV-1 *gag-protease*-driven replicative capacity influences cellular processes, including cytokine production and metabolism. We also identify inter-subtype differences in *gag-protease* driven RC, potentially illuminating the varied clinical outcomes observed in HIV-1 diseases across different subtypes and RCs. Future research should explore whether manipulating cytokines and cellular metabolism, in conjunction with other therapeutic or preventative approaches, could effectively limit viral transmission and improve clinical outcomes.

## MATERIALS AND METHODS

### Design

Participants for this study were selected from the previously described Sinikithemba and BC HOMER cohorts (5, 15). A total of 27 donors were included in this study based on the viruses’ phenotypic properties including the subtype and replicative capacity. The selection criteria for inclusion was having virus RC in the upper (high RC group) or lower (low RC group) 10% of the data set from the Sinikithemba cohort (*n* = 406, Durban, South Africa) and from the upper or lower quartile of the data set generated in Durban from the BC HOMER cohort (*n* = 24, British Columbia, Canada) (5, 15). The participants living with HIV-1 subtype C were predominantly female and slightly younger with higher CD4^+^ T-cell counts, however, this did not achieve statistical significance. Viral load setpoints were also similar in both subtype B and C study groups (Table 1). The same method was previously used for determining the virus RC for both cohorts (5, 15). Additional stored plasma samples of 21 participants from the subtype C HPP Acute Infection cohort with previously defined virus RC data, were utilized solely for circulating cytokine expression (38). The clinical and demographic characteristics are summarized in Table 2.

**TABLE 1:** Cross sectional clinical and demographic characteristics of antiretroviral therapy-naïve persons living with HIV-1 subtype B (BC Homer cohort) or subtype C (Sinikithemba cohort)

Characteristic^*a*^	HIV-1 subtype B infection group	HIV-1 subtype C infection group
No. of participants (% female)	12 (0%)	15 (87%)
Median (IQR) age of participants (years)	37.9 (32.1–48.0)	30.2 (26.4–39.3)
Median (IQR) CD4 count (no. of cells/μL)	220 (77.5–410)	347 (137-578)
Median (IQR) plasma viral load (log_10_ HIV RNA copies/mL)	5.4 (5.2–5.6)	5.2 (4.6–5.6)

^*a*^ IQR, interquartile range.

**TABLE 2:** Clinical and demographic characteristics of antiretroviral therapy-naïve persons with acute HIV-1 subtype C grouped based on the overall viral replicative capacity

Characteristic^*a*^	Low replicative capacity group	High replicative capacity group
No. of participants (% female)	11 (64%)	10 (80%)
Median (IQR) age of participants (years)	29 (25–39)	27 (24–36)
Median (IQR) CD4 count (no. of cells/μL)	361 (313–537)	399 (329–634)
Median (IQR) plasma viral load (log_10_ HIV RNA copies/mL)	5.15 (4.35–5.71)	5.52 (4.71–5.90)

^*a*^ IQR, interquartile range.

### Amplification, sequencing of HIV-1 *gag-protease* from plasma samples, and generation of Gag-protease recombinant viruses

HIV-1 *gag-protease* was amplified and sequenced using previously described protocols (5). Viral RNA was isolated from cryopreserved virus stocks (15) (group 1, subtype B viruses) or from donor plasma samples (group 2, subtype C viruses) using the Qiagen Viral RNA Mini kit (Qiagen, Hilden, Germany). HIV-1 *gag-protease* was amplified in a 2-step PCR including HIV-1 reverse transcription and *gag-protease* amplification using previously described primers (15) and sequenced by Sanger sequencing. The phylogenetic tree was created using Geneious version 2023.0 available from https://www.geneious.com. Recombinant viruses were generated by recombination of patient-derived HIV-1 *gag-protease* amplicons with linearized *gag-protease*-deleted NL4-3 plasmid, cotransfection into GFP-reporter CEM-GXR T-cells, and propagation in culture until 20% to 30% of the cells were infected as assessed on a FACSCalibur flow cytometer (BD Biosciences). CEM-GXR cells are an immortalized T-cell line engineered with a Tat-driven LTR-GFP expression cassette, thus, HIV-1 infection is identified by GFP expression (39). Thereafter, the culture supernatants were harvested and the virus stocks were stored at −80°C.

### Replicative capacity assay

Titration of the recombinant virus stocks and the RC assays were performed as previously reported (15). In brief, CEM-GXR T-cells were infected with a known volume of virus stock, and the frequency of GFP-expressing cells (infected GXR cells produce GFP) was quantified after 48 hours using flow cytometry. This value was used to calculate a multiplicity of infection of 0.003 for subsequent RC assays. For the RC assay, one million GXR T-cells were infected and monitored by flow cytometry for 6 days for GFP-positivity. The mean slope of growth for each virus (from days 3 to 6) was calculated using the semilog method in Excel and normalized to the growth of the wild-type NL4-3 control virus included in each assay. The RC experiments were performed in duplicate and averaged.

### Primary CD4^+^ T cell enrichment and infection

Primary peripheral blood mononuclear cells (PBMCs) were isolated from two healthy donors and enriched for CD4^+^ T cells using the CD4^+^ T Cell Isolation Kit (Miltenyi Biotech, Germany) according to the manufacturer’s protocols. Briefly, PBMCs were sequentially treated with the CD4^+^ T Cell Enrichment Cocktail and magnetic beads at 4°C for 5 minutes and 10 minutes, respectively. The sample was then passed through a MACS column placed in a magnetic field. Purified CD4^+^ T cells were collected from the flow-through and washes. Purity was assessed using flow cytometry, achieving >90% purity. Half a million CD4^+^ T cells were infected with 100 ng p24 equivalent of virus stocks via spinoculation (1,200 × *g* for 2 hours) and cultured for 72 hours. Supernatants were stored at −80°C until use in cytokine assays.

### Cytokine studies

Cytokine profiling studies of infected culture supernatants (harvested after 24 hours for CEM-GXR T-cells or 72 hours for primary CD4^+^ T cells) were performed in duplicate using the Bio-Plex human cytokine screening panel, 27-plex kit from Bio-Rad (Hercules, California, USA) according to the manufacturer’s instructions. The kit measures Eotaxin, FGF-basic, granulocyte colony-stimulating factor (G-CSF), granulocyte macrophage colony-stimulating factor (GM-CSF), interleukin (IL)-1β, IL-1 receptor antagonist (IL-1RA), IL-2, -4, -5, -6, -7, -8, -9, -10, -12, -13, -15, and -17, interferon-γ (IFN-γ), interferon gamma inducible protein (IP-10), MCP-1, MIP-1α, MIP-1β, PDGF-bb, regulated on activation, normal T cell expressed and secreted (RANTES), TNF-α and vascular endothelial growth factor. The data were collected using the Bio-Plex manager software version 6 on the Bio-Plex 200 plate reader (CA, USA). The manufacturer’s default 5PL regression formula was used to interpolate the concentration of cytokines in the samples from the standard curves. Cytokines that were expressed below the lower limit of detection were reported as half of the lowest reportable value for that cytokine.

### Gag p24 flow cytometry assay

Gag p24 flow cytometry was performed using previously described protocols (40). This entailed the fixation and permeabilization of cells using the Cytofix/Cytoperm (BD Biosciences) kit and intracellular staining with HIV Gag p24 RD1 antibody (clone KC57; Beckman Coulter, Indianapolis, IN, USA). Cells were acquired using an LSRFortessa (BD Biosciences) with FACSDiva software and the data were analyzed using the FlowJo version 10.0.8 (Flowjo, LLC, Ashland, OR).

### Cell-to-cell spread assay

The cell-to-cell spread ability of chimeric viruses was measured by modifying a previous protocol (41). Briefly, one million Jurkat cells were infected with 100 ng p24 equivalent of virus stocks by spinoculation (1,200 × *g*, 2 hours), and cultured for 72 hours to achieve up to 10% infectivity, which was quantified by the Gag p24 flow cytometry assay. These were designated as infected donor cells and labeled with cell proliferation eFlour450 dye (ThermoFisher Scientific, Massachusetts). Fresh Jurkat cells, designated as target cells were labeled using the CellTrace Far red cell proliferation kit (ThermoFisher Scientific, USA) and co-cultured 1:1 with infected donor cells for up to 48 hours (0.5 × 10^6^ cells in total). HIV-1 cell-to-cell spread was measured after 24 and 48 hours by Gag p24 detection within target cells using an LSRFortessa (BD Biosciences) with FACSDiva software. Results were analyzed using FlowJo version 10.0.8 (Flowjo, LLC, Ashland, OR). Cell culture supernatants were collected at 24 hours and stored at −80°C for glutamine quantification.

### Glutamine quantification

Glutamine levels within culture supernatants were quantified in a 2-step assay using the bioluminescent glutamine/glutamate kit (Promega Corporation, Madison, WI, USA) according to manufacturer instructions as follows. Dilutions (1:10) of culture supernatants were plated in quadruplicate in a 96-well plate. Two of the four wells were used for quantifying basal levels of glutamate (no glutaminase enzyme was added), while the other two wells were used to measure total glutamate levels following the addition of glutaminase which converts glutamine to glutamate. The luminescent signal is proportional to the amount of glutamate in each well. Concentrations were extrapolated using a standard curve using the sigmoidal 4PL curve fit in Graphpad Prism v 9.4 software (La Jolla, CA, USA). Glutamine levels were indirectly determined by subtracting the average reading for the “no glutaminase enzyme wells” (background glutamate) from the wells that included the glutaminase enzyme (background glutamate and glutamate converted from glutamine).

### Analysis of glucose uptake, fatty acid uptake, and mitochondria polarization

Virus infectivity was achieved by resuspension of Jurkat cells in a medium containing 100 ng p24 equivalent of virus stocks followed by centrifugation at 1,200 × *g* for 2 hours at room temperature. The supernatant was decanted and the cells were resuspended in fresh medium and incubated for 72 hours at 37°C with 5% CO_2_ to establish infection. Glucose uptake, fatty acid uptake, or mitochondrial function of infected Jurkat cells was measured by incubating cell cultures with either 2-[N*-*(7*-*nitrobenz-2-oxa-1,3-diazol-4-yl)amino]-2-deoxyglucose (2-NBDG [100 µM], ThermoFisher Scientific), Bodipy (12.5 µM, ThermoFisher Scientific) or a combination of mitotracker green (MTG, 100 nM, ThermoFisher Scientific) and mitotracker deep-red dyes (MTDR; 12.5 nM, ThermoFisher Scientific) for 30 minutes at 37°C as per a previous protocol (42). Cells were washed and stained for intracellular Gag p24 and acquired using the LSR-Fortessa and the Diva software (BD, Biosciences). Data were analyzed using FlowJo version 10.7.1 (BD). Mitochondrial depolarization was determined using the combination gates function to identify the frequency of cells positive for the MTG but not MTDR.

### Data analysis

Cytokine data were analyzed using uniform manifold approximation and projection (UMAP) for dimensionality reduction and visualization of high-dimensional data. Group comparisons were performed using the Student’s *t*-test and if assumptions of the test were not met, the non-parametric Mann-Whitney *U* test was applied. Correlations between variables were assessed using Pearson’s correlation test or Spearman’s rank correlation test, depending on data distribution. Statistical significance was defined as *P* < 0.05. Data analysis was conducted using Graphpad Prism version 9.4 software and R studio.

## Data Availability

The nucleotide sequences are available in GenBank under accession numbers EU242168.1, EU242207.1, EU242334.1, EU242344.1, EU242384.1, EU242434.1, EU242394.1, EU242429.1, EU241942.1, HM593138.1, HM593483.1, HM593489.1, HM593141.1, HM593172.1, HM593179.1, HM593182.1, HM593195.1, HM593243.1, HM593279.1, HM593313.1, HM593362.1, HM593443.1, HM593462.1, and HM593271.1.

## SUPPLEMENTAL MATERIAL

Figure S1 mbio.03565-24-s0001.eps Impact of HIV-1 RC and subtypes on cytokine expression in T cells.

Figure S2 mbio.03565-24-s0002.eps Impact of HIV-1 RC and subtypes on cell-to-cell spread and glutamine consumption in T cells.

Figure S3 mbio.03565-24-s0003.eps Impact of HIV-1 RC and subtypes on nutrient uptake and mitochondrial function in T cells.

Supplemental Legends mbio.03565-24-s0004.docx Legends for Fig. S1 to S3.

ASM does not own the copyrights to Supplemental Material that may be linked to, or accessed through, an article. The authors have granted ASM a non-exclusive, world-wide license to publish the Supplemental Material files. Please contact the corresponding author directly for reuse.
