# Supplementary material for: HIV-1 Gag-protease-driven replicative capacity influences T-cell metabolism, cytokine induction, and viral cell-to-cell spread
Source: mBio. 2025 Feb 25;16(4):e03565-24. doi: 10.1128/mbio.03565-24 (PMC11980368; doi:10.1128/mbio.03565-24)
Supplement: Supplemental Legends — Legends for Fig. S1 to S3. [file mbio.03565-24-s0004.docx]

**Supplementary Figure legends**

**Supplementary Figure 1: Influence of HIV-1 subtypes and replication capacities (RCs) on cytokine expression in GXR-T, primary CD4+ T cells and in plasma from acute HIV-1 infection.** (a) Representative cytokine levels, including IL-7, PDGF-bb, FGF-basic, and MCP-1, in GXR-T cells across different study groups and controls. (b) Summary of IL-7, PDGF-bb, FGF-basic, and MCP-1 levels in primary CD4+ T cells. (c) Distribution of low versus high RCs among selected participants from an HIV-1 subtype C infection cohort. (d) Comparison of PDGF-bb and IL-7 levels in plasma samples from individuals with HIV-1 subtype C infection. All statistical tests are two-sided. Mann-Whitney U test P values are shown in panels (a, b, c, d).

**Supplementary Figure 2: Impact of HIV-1 Gag-protease mediated replication capacities (RCs) on virus cell-to-cell spread and glutamine consumption.** (a) Comparison of the frequency of Gag p24-positive donor cells at the start (0 hours) and target cells after 24 hours of co-culture across study groups and controls. (b) Correlation between virus RCs and the percentage increase in Gag p24-positive target cells from p24-positive donor cells after 24 hours of co-culture. (c) Glutamine levels in virus-infected cell culture supernatants after 24 hours. Two-tailed *P* values from Mann-Whitney U (a) and Kruskal-Wallis (c) tests are displayed. Spearman’s rho (*r*) values and *P* values are reported for the correlation analysis (b).

**Supplementary Figure 3: Impact of HIV-1 Gag-protease mediated replication capacities (RCs) on T-cell nutrient uptake and mitochondrial function.** (a) Aggregate data for glucose uptake measured in infected cultures and controls. (b-d) Aggregate data for fatty acid uptake measured in total live cells (b) and in infected cultures and controls. Summary data for total fatty acid uptake in all live cells, bystander uninfected (p24-negative; neg) (c), and infected (p24-positive; pos) (d) cells. (e) Comparison of mitochondrial mass, measured as the percentage of Mitotracker Green (MTG)-positive live cells, across RC groups and controls. (f) Comparison of mitochondrial mass across subtype groups. (g) Aggregate data comparing mitochondrial membrane depolarization across subtype groups. Two-tailed *P* values from the Mann-Whitney U test are displayed.
